# Biomechanical insights into increasing jump distance in ski jumping: a review classified by hill size

**DOI:** 10.3389/fspor.2026.1784281

**Published:** 2026-06-19

**Authors:** Yuta Funato, Shinji Sakurai

**Affiliations:** 1 Graduate School of Health and Sport Sciences, Chukyo University, Toyota, Japan; 2 School of Health and Sport Sciences, Chukyo University, Toyota, Japan

**Keywords:** approach, takeoff, flight, landing, coaching

## Abstract

This review organizes biomechanical studies aimed at increasing the jump distance in ski jumping by phase (approach, takeoff, flight, and landing) and classifies them according to hill size. Data were collected from EBSCOhost, Web of Science, and Google Scholar. A total of 1,701 records were retrieved from the databases, of which 42 met the inclusion criteria and were included in this review. At first glance, the literature appears to comprehensively cover findings that meet the mechanical objectives required to increase jump distance in each phase. However, when examined by hill size, the majority of literature is concentrated on normal and large hills, which are used in the Olympic Games. Consequently, there is limited scientific evidence regarding small-scale hills (small and medium hills) and largest-scale hills (flying hills). Therefore, future studies should focus on hills other than normal and large hills. To facilitate the application of kinematic data to musculoskeletal models and the estimation of kinetics, there is a need to develop methods that enable easy recording of jumpers and collection of kinematic data with minimal error. Furthermore, because research on landing remains insufficient, further studies on the landing phase are required from an injury-prevention perspective.

## 1 Introduction

Ski jumping is scored based on both jump distance and flight style, and jumpers are ranked according to their total score. As distance points account for a large proportion of the overall score, jump distance has a substantial impact on competitive performance. Consequently, jumpers primarily aim to increase their jump distance. Therefore, numerous biomechanical studies have focused on the factors related to increasing the jump distance.

The earliest study is often attributed to Straumann (1), who addressed body posture during the flight phase (2). Since then, biomechanical studies have accumulated in parallel with improvements in jumping technique, rule revisions, and advances in equipment. A representative example of technical development is the establishment of the V-style technique, in which skis are spread in a V-shape during the flight phase. In regard to competition rules, a notable change concerns the determination of ski length. The former rule, based on adding 80 cm to body height, was replaced by a proportional rule (currently 145%). In addition, to prevent jumpers from adopting excessively low body mass, a rule was introduced whereby the allowable ski length ratio varies according to the body mass index (BMI). Even today, technical improvements and rule revisions continue to occur. Thus, in ski jumping, rules have been revised in accordance with improvements in jumper techniques to ensure the fairness of competition and protect jumper health. It is desirable that such rule changes be established based on objective biomechanical evidence.

In fact, Janura et al. (3) and Janurová et al. (4) investigated changes in the initial position over the past decade as well as the relationship between approach velocity and jump distance. In particular, Janura et al. (3) reported that although approach velocity decreased, jump distance increased and attributed this outcome to advances in technique and equipment. Along with these developments, biomechanical studies on ski jumping continued to accumulate, and two major reviews have been published to date (5, 6). Schwameder and Müller (5) classified biomechanical studies up to 2001 based on kinematics and kinetics and presented the main findings. Schwameder (6) reviewed biomechanical studies conducted between 1991, when the V-style technique was introduced, and 2006. The studies were categorized according to the analysis methods (e.g., two-dimensional or three-dimensional) and measurement techniques (e.g., force plates or plantar pressure systems). In addition, the studies were classified into categories such as kinematics, ground reaction force (GRF), electromyography (EMG), computer simulation, aerodynamic forces, and others, and their respective proportions were reported.

**Figure 1:**
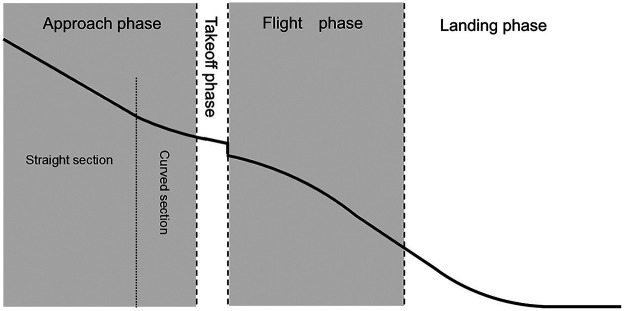
Definition of phases. The solid line represents the side profile of the ski jumping hill. The dotted lines indicate the moments at which the phases change. The transition point from the flight to landing phase varies depending on jump distance.

Ski jumping can be divided into four phases: approach, takeoff, flight, and landing (Figure 1). This classification is based on the differences in the forces acting on the jumper-ski system in each phase.

This review first explains the classification of ski jumping hill sizes. Subsequently, findings obtained from previous studies are organized according to each phase, with particular emphasis on biomechanical insights related to increasing jump distance. Simultaneously, the reviewed studies are limited to those conducted using actual jumps on ski jumping hills or under conditions closely resembling actual jumps and they are classified according to hill size. Finally, perspectives for future studies on ski jumping are presented by focusing on hill size.

## 2 Classification of hill sizes

The size of the ski jumping hills is defined by the rules of the International Ski and Snowboard Federation (FIS), as shown in Table 1 (7). As indicated in Table 1, ski-jumping hills are classified based on hill size. The hill size is defined as the maximum distance at which an elite jumper can safely land in an upright position (8). In competitions, the start gate is set and managed so that the jumpers do not exceed the hill size.

**Table 1:** Classification of hill sizes.

Classification	Hill Size (HS)	(Note) Distance of w
Small hill	49 m and under	44 m and under
Medium hill	50 m to 84 m	45 m to 75 m
Normal hill	85 m to 109 m	76 m to 98 m
Large hill	110 m to 145 m	99 m to 130 m
Flying hill	185 m and over	166 m and over

Recently, giant hills have been added as a category to fill the gap between large and flying hills (7). However, currently, no World Cup competitions are held on giant hills, and no previous studies have specifically focused on them. Therefore, giant hills were not included in the classification of ski jumping hill sizes used in this review.

**Figure 2:**
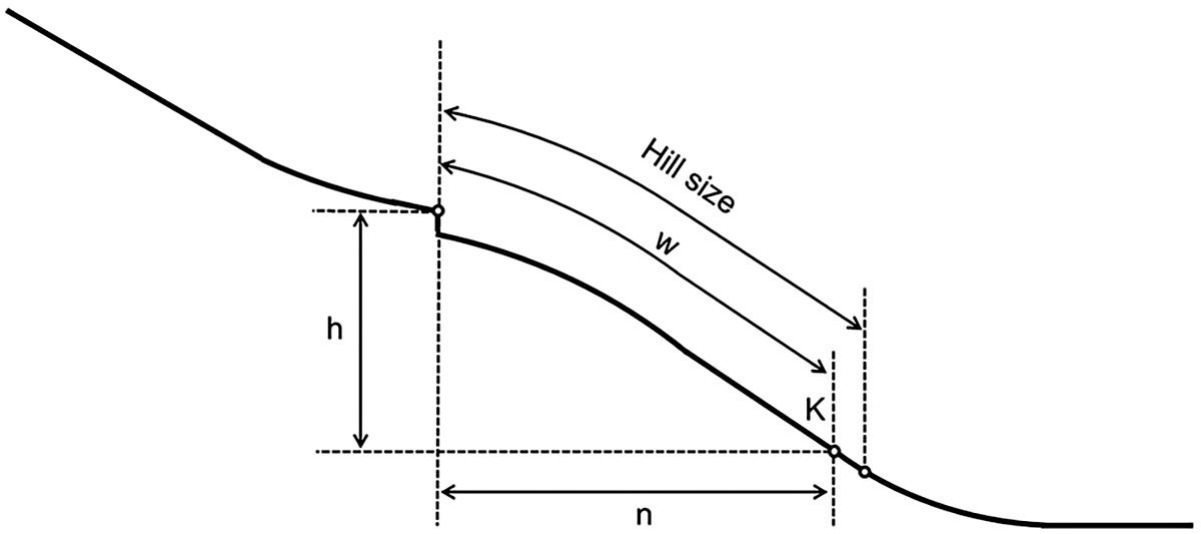
Hill profile parameters involved in determining hill size. The thick solid line represents the side profile of the ski jumping hill. K, K-point; w, distance between the end of the in-run and the K-point; h, vertical distance between the end of the in-run and the K-point; n, horizontal distance between the end of the in-run and the K-point.

Figure 2 illustrates the hill profile elements involved in determining hill size and is used to define hill size.

Hill size is calculated based on the distance w used in the construction of the ski jumping hill, as shown in Table 1 (8). Distance w is defined as the distance between the end of the approach section of the ski jumping hill (in-run) and the K-point. In ski jumping hill design, the allowable range of the ratio between vertical (h) and horizontal distance (n) from the end of the in-run to the K-point (h/n) is specified. For example, when the K-point is 90 m, the h/n ratio is defined to be approximately between 0.55 and 0.50. When converted into actual distances, this corresponds to an h range of approximately 40–43 m and n range of approximately 79–81 m.

As a result, as the size of the ski jumping hill increases, the jumper's velocity inevitably increases. For example, the approach velocity on a medium hill is approximately 80 km/h, whereas that on a flying hill is approximately 100 km/h. Changes in speed alter the magnitude of the forces acting on jumpers. As the hill size increases, the velocity increases. This results in relatively greater aerodynamic forces acting on the jumper, thereby requiring a higher-level technique. In addition, as the hill size increases, the jump distance increases. Therefore, the contribution of the technique during the flight phase has become more important as the hill size has increased. Flight time has been shown to be longer on large hills than on normal hills (9).

In addition, when normal and large hills are constructed at the same site, they must be built with a difference in hill size of at least 25 m (7). In Japan, the only site where a normal hill and a large hill are colocated is Hakuba Village in Nagano Prefecture, with hill sizes of 98 m and 134 m, respectively. Therefore, it is not realistic for jumpers to adjust the hill size they use in small increments. In other words, when a jumper switches from one hill category to another, the sensation differs markedly from that experienced on the previous hill. Note that, in addition, the profile of each ski jump varies slightly, such as in the angle of the in-run.

## 3 Objectives, hypotheses, and search strategy

Based on these considerations, it is possible that jumpers adopt different strategies depending on hill size. Therefore, organizing existing biomechanical findings according to hill size is useful for examining such strategic differences. This review aimed to confirm whether biomechanical insights related to an increase in ski jumping distance have been comprehensively reported across all hill sizes. By doing so, it becomes possible to identify hill sizes for which knowledge is limited and clarify those that require further research. Increasing the knowledge of underrepresented hill sizes would contribute to improving jumper strategies and coaching practices, particularly for enhancing the performance of junior jumpers.

In ski jumping, Olympic events are limited to normal hills and large hills, and World Cup competitions are predominantly held on large hills. Therefore, it is reasonable to assume that many previous studies have focused on normal and large hills.

**Figure 3:**
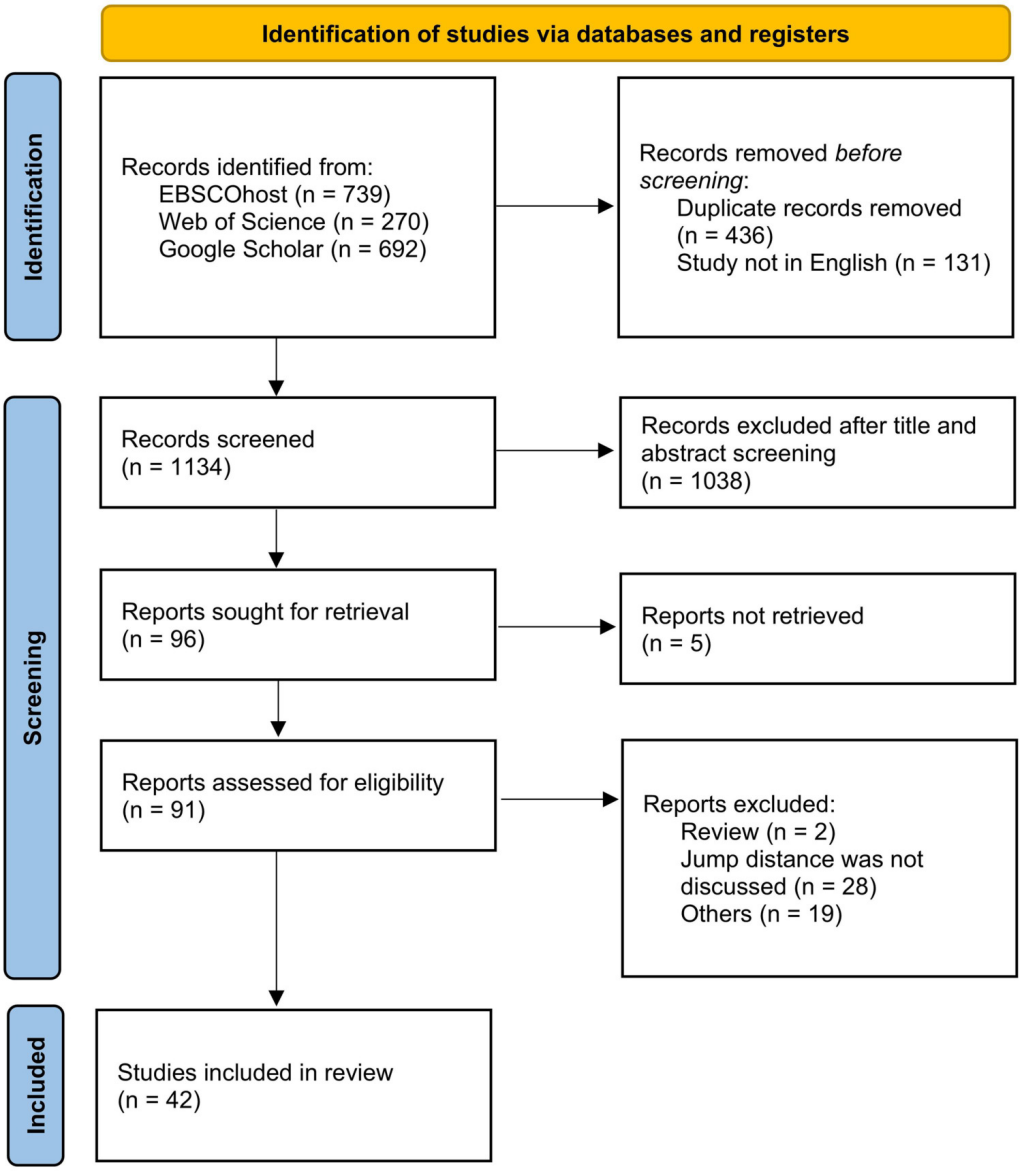
PRISMA flow diagram.

For this review, relevant literature was collected from EBSCOhost, Web of Science, and Google Scholar. The relevant search terms were applied to titles and included combinations of “ski jump,” “ski jumping,” “ski jumper,” “ski jumpers,” and “ski jumper's.” A total of 1,701 records were initially identified from the databases. After removing duplicates, 1,134 records remained. The inclusion criteria were peer-reviewed articles that described relationships with jump distance. The exclusion criteria included non-peer-reviewed articles, reviews, conference abstracts, books, non-English publications, studies not describing relationships with jump distance, studies not related to biomechanics, and studies unrelated to ski jumping. Ultimately, 96 articles were reviewed, of which 42 were included (Figure 3). All the selected studies are included in the tables of this review.

## 4 Findings in each phase and classification of previous studies by hill size

### 4.1 Approach phase

#### 4.1.1 Mechanical objectives for increasing jump distance

The approach phase consists of descending a slope of approximately −35°, followed by a curved section with a radius of approximately 100 m. In recent years, some ski jumping hills have been constructed using a clothoid curve for the curved section (7, 8).

**Figure 4:**
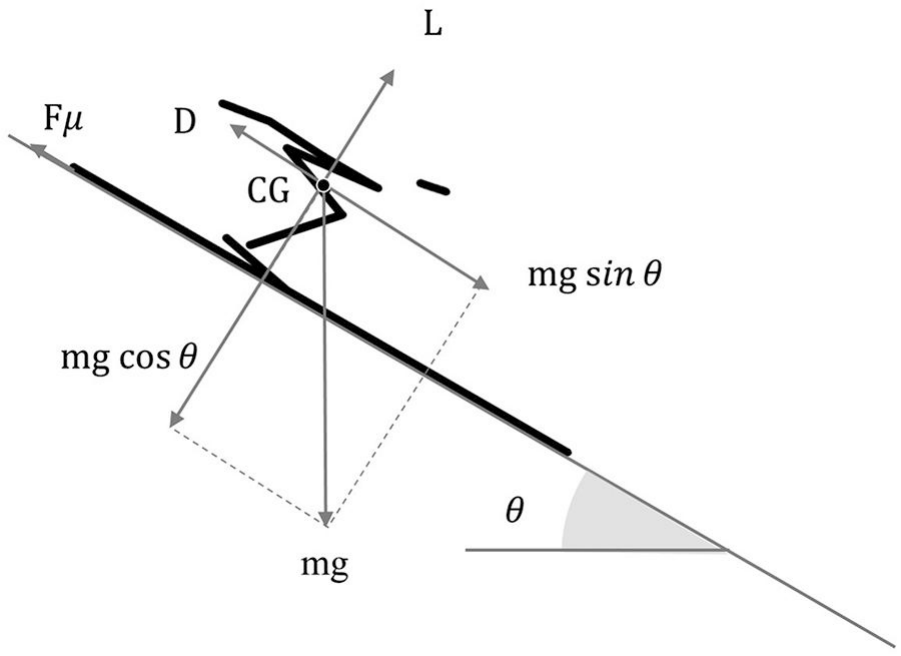
Forces or force components acting on the jumper-ski system during the straight section of the approach phase. *θ*, Angle of the approach section of ski jumping hill at the straight section of the approach phase; mg, Gravity; L, Lift; D, Drag; F*μ*, Friction force; CG, Center of gravity of the jumper-ski system.

**Figure 5:**
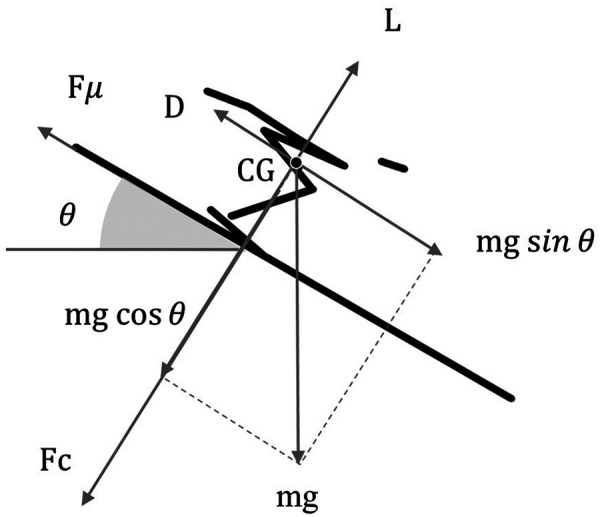
Forces or force components acting on the jumper-ski system during the curved section of the approach phase. *θ*, Angle of the approach section of ski jumping hill at the curved section of the approach phase; mg, Gravity; L, Lift; D, Drag; Fμ, Friction force; CG, Center of gravity of the jumper-ski system; Fc, Centrifugal force.

The approach phase can be divided into straight and curved sections (Figure 1). The forces or force components acting on the jumper-ski system in the straight section of the approach phase are shown in Figure 4. Similarly, the forces or force components acting on the jumper in the curved section of the approach phase are illustrated in Figure 5. The objective of the approach phase is to acquire a high approach velocity. Therefore, in order for the jumper to increase the approach velocity, it is necessary to reduce aerodynamic drag. Since the drag depends on the projected area perpendicular to the direction of motion, the jumper must adopt a low initial position. In addition, increasing the body or equipment mass increases the component of gravitational force acting in the direction of motion. The friction between the skis and snow surface also affects the approach velocity. Thus, reducing drag, increasing mass, and minimizing friction are necessary to achieve a higher approach velocity. However, mass also affects gravitational force during the flight phase. In other words, mass represents a trade-off between the approach and flight phases. Although the influence of friction force is relatively small, it becomes important at higher competitive levels. Most importantly, reducing drag is essential. Minimizing drag during the approach phase increases approach velocity and therefore consistently contributes to an increase in jump distance in the subsequent phases as well.

#### 4.1.2 Approach velocity, drag, initial position, and mass

In actual jumps, it has been reported that an increase in approach velocity increases jump distance on medium hills (10). Similarly, on normal hills, the importance of acquiring a high velocity during the initial position and minimizing the reduction of the acquired approach velocity has been demonstrated (9, 11–14).

Elfmark and Ettema (15) used computer simulations to demonstrate that a reduction in the projected area perpendicular to the direction of motion led to a higher approach velocity. In addition, several studies reported that lower initial positions are associated with longer jump distances (15, 16). Computer simulations are based on specific models, whereas the body shapes of the actual jumpers vary. Therefore, there are limitations to generalizing the findings of such studies, particularly for junior jumpers.

In the curved section, as shown in Figure 5, centrifugal force acts on the jumper-ski system. Ettema et al. (17) investigated curved sections using computer simulations and demonstrated that jumpers maintain their position by increasing the joint torques when transitioning into a curved section. This suggests that, if the position cannot be maintained, the projected area may increase, thereby increasing the aerodynamic drag.

Some studies on actual jumps have reported a positive correlation between mass and approach velocity (12). Although it is evident that an increase in mass increases velocity, jumpers generally aim to reduce their body mass as much as possible. In recent years, rules have been introduced whereby ski length is reduced if the BMI falls below a certain threshold. However, some jumpers still reduce their body mass even at the expense of ski length. Nevertheless, it is likely that, due to the BMI regulations, the number of jumpers with extremely low body mass has decreased in recent years.

#### 4.1.3 Friction

Kim et al. (18) investigated the friction force and reported that the coefficient of kinetic friction between the jump skis and snow surface was approximately 0.08. The importance of ski wax and ski base structure (fine grooves on the sliding surface) was also highlighted.

#### 4.1.4 Classification of previous studies by hill size

Previous studies on the initial position, conducted either as actual jumps or under conditions similar to actual jumps, can be classified according to the hill size, as shown in Table 2. Although most of the available findings are derived from studies conducted on normal hills, they consistently demonstrate the importance of acquiring the approach velocity during the initial position. In addition, strategies for increasing the approach velocity have also been discussed. Considering the low coefficient of friction and the effects associated with increased mass during the flight phase, adopting an efficient approach position in the approach phase is useful for increasing the jump distance. However, because most previous studies have focused on normal hills, obtaining findings from the smallest scale (small hill) and largest scale (flying hill) would help clarify the overall trends.

**Table 2:** Main focuses of previous studies in the approach phase and classification by hill size.

Previous study	Hill Size					Main focus						
	Small hill	Medium hill	Normal hill	Large hill	Flying hill	Relationship between approach velocity and jump distance	Mass	Drag	Initial position	Friction between skis and snow	Main findings	Notes
Virmavirta and Komi (10)	No	Yes	No	No	No	Yes	No	No	No	No	An increase in approach velocity increases jump distance.	
Komi et al. (11)	No	No	Yes	No	No	Yes	No	No	No	No	An increase in approach velocity increases jump distance.	
Virmavirta and Komi (12)	No	No	Yes	No	No	Yes	Yes	No	No	No	An increase in approach velocity increases jump distance.	
Virmavirta et al. (13)	No	No	Yes	No	No	Yes	No	No	No	No	An increase in approach velocity increases jump distance.	
Vodicar and Jost (14)	No	No	Yes	No	No	Yes	No	No	No	No	An increase in approach velocity increases jump distance.	
Zanevskyy and Banakh (16)	No	Yes	No	No	No	No	No	No	Yes	No	Jumpers who achieved longer jump distances had a lower initial position.	
Elfmark and Ettema (15)	No	No	Yes	Yes	No	No	No	Yes	No	No	A reduction in the projected area of the approach position relative to the direction of motion increases approach velocity.	
Ettema et al. (17)	No	No	Yes	Yes	No	No	No	Yes	No	No	Jumpers maintain their position in the curved section by increasing lower-limb joint torques.	This study focuses on the curve section only.
Kim et al. (18)	Yes	Yes	Yes	Yes	Yes	No	No	No	No	Yes	The coefficient of kinetic friction between the ski jumping skis and the snow surface is approximately 0.08.	

Yes, Indicates that it is subject; No, Indicates that it is not subject.

### 4.2 Takeoff phase

#### 4.2.1 Mechanical objectives for increasing jump distance

The profile of the ski jumping hill in the takeoff phase consists of a straight section of approximately 5.5 m with an inclination of about −9.5° (takeoff table).

The takeoff phase is considered particularly important because it directly affects the initial state of the subsequent flight phase (6, 19). Additionally, two mechanical objectives for increasing jump distance during the takeoff phase were identified (6). The first objective is to acquire vertical velocity of the center of gravity (CG) of the jumper-ski system at takeoff (19). The second objective is to acquire an appropriate amount of forward angular momentum about the CG of the jumper-ski system at takeoff (20, 21). Additionally, maintaining the horizontal velocity of the jumper-ski system is important (19).

**Figure 6:**
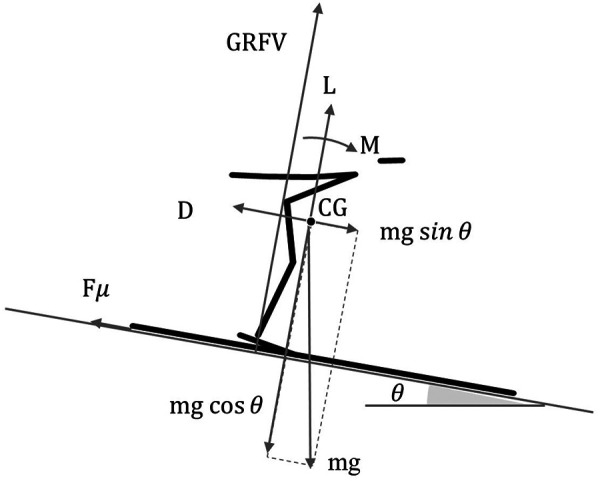
Forces and force components acting on the jumper-ski system during the takeoff phase. *θ*, angle of the approach section of ski jumping hill at takeoff phase; mg, Gravity; L, Lift; D, Drag; Fμ, Friction force; GRFV, Ground reaction force vector; M, Moment; CG, Center of gravity of the jumper-ski system.

The forces or force components acting on the jumper-ski system during the takeoff phase are shown in Figure 6.

To achieve these two mechanical objectives in the takeoff phase, jumpers perform the takeoff motion. The takeoff motion during ski jumping is performed under unique conditions. First, wearing ski boots restricts the range of motion of the lower limb joints (22). In addition, the takeoff motion is executed within a very short duration of approximately 0.25–0.3 s (23). During this period, the coefficient of friction between the skis and snow surface is small, and the friction force is therefore limited (6). In other words, the takeoff motion must be performed on a takeoff table, where the frictional force is nearly zero. Under this condition, the ground reaction force vector (GRFV) acts only in the direction perpendicular to the takeoff table. As the jumper slides down a downward slope and the ankle joint is fixed in a flexed position, performing a typical jumping motion prevents plantar flexion of the ankle, causing the jumper to fall forward.

Notably, the length and inclination of the takeoff table differ among ski jumping hills, and depending on the jumper, the takeoff motion may begin in the latter part of the curved section of the approach phase. The radius of the curved section is approximately 100 m for large hills and about 35 m for small hills; therefore, its influence on the CG of the jumper-ski system at the onset of the takeoff motion is considered to be limited.

#### 4.2.2 The vertical velocity of the CG of the jumper-ski system

In ski jumping, the velocity and velocity vector of the CG of the jumper-ski system at takeoff can be considered the initial conditions of projectile motion.

**Figure 7:**
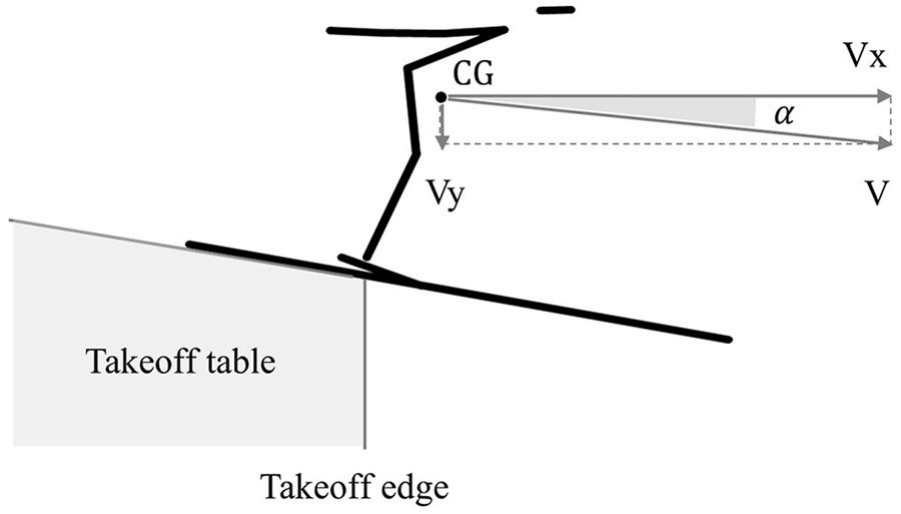
Velocity vector and its components of the center of gravity of the jumper-ski system at takeoff. V, Velocity vector; Vx, Horizontal component of the velocity vector; Vy, Vertical component of the velocity vector; *α*, Angle between the velocity vector and the horizontal axis; CG, Center of gravity of the jumper-ski system.

Studies based on actual jumps have shown that, on large hills, the angle of attack of skis at takeoff is approximately −5° (24, 25). In addition, studies on medium hills have shown that the angle of the velocity vector of the CG of the jumper-ski system at takeoff is approximately −4° relative to the horizontal axis. In other words, the velocity vector at takeoff during ski jumping is directed downward (Figure 7). Therefore, during the takeoff motion, the jumper is required to reduce the vertical component of velocity vector (Vy) and angle (*α*) between velocity vector and horizontal axis.

To acquire vertical velocity of the CG of the jumper-ski system, it is necessary to increase the impulse of the GRFV. In addition, the lift also contributes to this process.

Positive correlations have been reported between the impulse of the GRFV during takeoff motion, vertical velocity of the CG of the jumper-ski system obtained from video analysis, and jump distance (10, 11, 14). In contrast, a previous study conducted on a large hill (25) reported a significant negative correlation between the vertical velocity of the CG of the jumper-ski system immediately after takeoff and jump distance. Together, these findings suggest that as hill size decreases, the influence of the vertical velocity of the CG of the jumper-ski system on the jump distance becomes stronger. Furthermore, Virmavirta et al. (25) showed that in highly skilled jumpers, a negative correlation was observed between the vertical velocity of the CG of the jumper-ski system immediately after takeoff and jump distance. This was associated with the acquisition of approach velocity. Therefore, in jumpers with a high competitive level, a takeoff motion that suppresses the reduction of approach velocity may contribute to an increase in jump distance. However, whether the observed negative correlation is due to increasing hill size or differences in jumper skill level remains unclear. In particular, because large hills and flying hills can only be attempted by highly skilled jumpers, clarifying this issue is currently difficult.

Virmavirta et al. (26) demonstrated through wind tunnel experiments that the duration of the takeoff motion decreased as the wind speed increased. However, this study also showed that the reduction in GRF associated with the shorter takeoff duration was compensated for by lift, indicating that the aerodynamic effects were not sufficient for the jumper to gain additional lift compared with calm conditions.

#### 4.2.3 Angular momentum

Immediately after the takeoff motion, the jumper transitions into the flight position. To transition smoothly into the flight position, it is necessary to acquire the forward angular momentum about the CG of the jumper-ski system at takeoff. There are three possible strategies for generating the forward angular momentum for the CG of the jumper-ski system. The first strategy is to increase the distance between the CG of the jumper-ski system and GRFV, while GRFV passes behind CG. The second strategy is to increase the impulse of the GRFV as it passes behind the CG of the jumper-ski system. The third strategy is to achieve both simultaneously.

In actual jumps, Funato et al. (21) calculated angular momentum on a medium hill. That study's participants included a range from junior jumpers to Olympic-level jumpers. It was shown that the relationship between angular momentum at takeoff and jump distance can be approximated by a quadratic function. However, two reasons were proposed for this relationship. First, excessive acquisition of forward angular momentum may lead to a fall. Second, the generation of angular momentum may negatively affect the vertical velocity of the CG in the jumper-ski system. However, the specific strategies for acquiring angular momentum were not clarified.

As Funato et al. (21) is the only study that has clearly quantified angular momentum, the applicability of this finding to other hill sizes remains unclear.

#### 4.2.4 Takeoff motion and reduction of drag

Many studies have investigated takeoff motion (10–13, 27). These studies indicate that the fundamental takeoff motion involves maintaining a small shank angle, extending the knee joint, and reducing the trunk angle at takeoff. This basic movement pattern is considered to be a common finding regardless of hill size. These movements are also thought to minimize the projected area as much as possible. In practice, Yamamoto et al. (28) and Hu et al. (29) investigated drag using computer simulations. These studies demonstrated that an increase in the projected area leads to an increase in drag.

More detailed investigations of the takeoff motion have also been conducted. Some studies have examined plantar pressure and EMG during actual jumps (30, 31). Virmavirta et al. (34) demonstrated that plantar pressure and EMG patterns remained nearly constant regardless of hill size. In contrast, Huang et al. (32) conducted a study using a musculoskeletal model and reported that consistent activation patterns were not observed in many muscles when compared with previous EMG findings. Huang et al. (32) used a proprietary system that automatically recognizes jumper motion from a markerless video. Their findings suggest that this discrepancy may be due to measurement error while also indicating limitations in evaluating musculoskeletal models using this method. Furthermore, it has been reported that the takeoff motion of highly skilled jumpers exhibits substantial variability (13, 33).

#### 4.2.5 Friction

The frictional force during the takeoff phase is determined by the coefficients of kinetic friction and vertical resistive force. The GRFV during the takeoff motion instantaneously reached approximately 1,200 N (34). In contrast, the coefficient of kinetic friction of skis is approximately 0.08 (18). Therefore, the friction force resulting from the normal reaction force during takeoff can be considered negligible.

#### 4.2.6 Classification of previous studies by hill size

The studies on the takeoff phase based on actual jumps or conditions similar to actual jumps are classified according to hill size and the results are summarized in Table 3. In the takeoff motion, it can be stated that during the takeoff motion it is important to acquire an appropriate amount of angular momentum while maximizing the vertical velocity at takeoff. However, studies on angular momentum are limited, and the applicability of these findings to hills of other sizes remains unclear. Therefore, further research on angular momentum in the takeoff phase is particularly needed for hill sizes other than medium.

**Table 3:** Main focuses of previous studies in the takeoff phase and classification by hill size.

Previous study	Hill Size					Main focus				
	Small hill	Medium hill	Normal hill	Large hill	Flying hill	Vertical velocity of the center of gravity of the jumper-ski system	Aerodynamic forces	Angular momentum	Friction between skis and snow	Takeoff motion (including plantar pressure and electromyography)
Komi et al. (11)	No	No	Yes	No	No	Yes	No	No	No	Yes
Virmavirta and Komi (33)	No	Yes	No	No	No	No	No	No	No	Yes
Vodicar and Jost (14)	No	No	Yes	No	No	Yes	No	No	No	No
Virmavirta et al. (25)	No	No	No	Yes	No	Yes	No	No	No	No
Virmavirta et al. (26)	No	Yes	Yes	Yes	Yes	No	Yes	No	No	No
Funato et al. (21)	No	Yes	No	No	No	No	No	Yes	No	No
Virmavirta and Komi (12)	No	No	Yes	No	No	No	No	No	No	Yes
Virmavirta and Komi (10)	No	Yes	No	No	No	Yes	No	No	No	No
Arndt et al. (27)	No	No	Yes	No	No	No	No	No	No	Yes
Virmavirta et al. (13)	No	No	Yes	No	No	No	No	No	No	Yes
Virmavirta and Komi (30)	No	No	Yes	No	No	No	No	No	No	Yes
Virmavirta et al. (31)	Yes	Yes	Yes	No	No	No	No	No	No	Yes
Yamamoto et al. (28)	No	No	Yes	No	No	No	Yes	No	No	No
Hu et al. (29)	No	No	Yes	No	No	No	Yes	No	No	No
Kim et al. (18)	Yes	Yes	Yes	Yes	Yes	No	No	No	Yes	No

Yes, Indicates that it is subject; No, Indicates that it is not subject.

#### 4.2.7 Findings related to training of the takeoff motion

Due to the nature of ski jumping, the number of training jumps performed as actual jumps is limited. Typically, the maximum number is around a dozen jumps per day. In addition, training may not be possible depending on the weather conditions, such as strong winds. Therefore, training methods known as simulation jumps, which aim to improve takeoff technique on the ground without performing actual jumps, are widely used.

Simulation jumps are performed under various conditions, such as using training shoes or performing jumps on boards equipped with rollers that eliminate friction at the sole of the foot. In some cases, simulation jumps are also performed while wearing ski jumping boots. However, as described above, the takeoff motion in actual jumps is performed under highly specific conditions. Therefore, studies focusing on simulation jumps have been conducted (22, 35–40). These studies have shown that, among simulation jumps, those performed on boards equipped with rollers that eliminate friction at the sole of the foot are the most similar to actual jumps. Lorenzetti et al. (38) demonstrated that simulation jumps performed on a board equipped with rollers exhibit kinematics similar to those of actual jumps. However, in actual ski jumping, aerodynamic forces are also present. As noted above, the takeoff motion is influenced by these aerodynamic forces; therefore, the inability to account for them represents a limitation.

### 4.3 Flight phase

#### 4.3.1 Mechanical objectives for increasing jump distance

When considered as a particle system, the jump distance is determined by the initial conditions at takeoff and the forces acting on the CG of the jumper-ski system during the flight phase.

**Figure 8:**
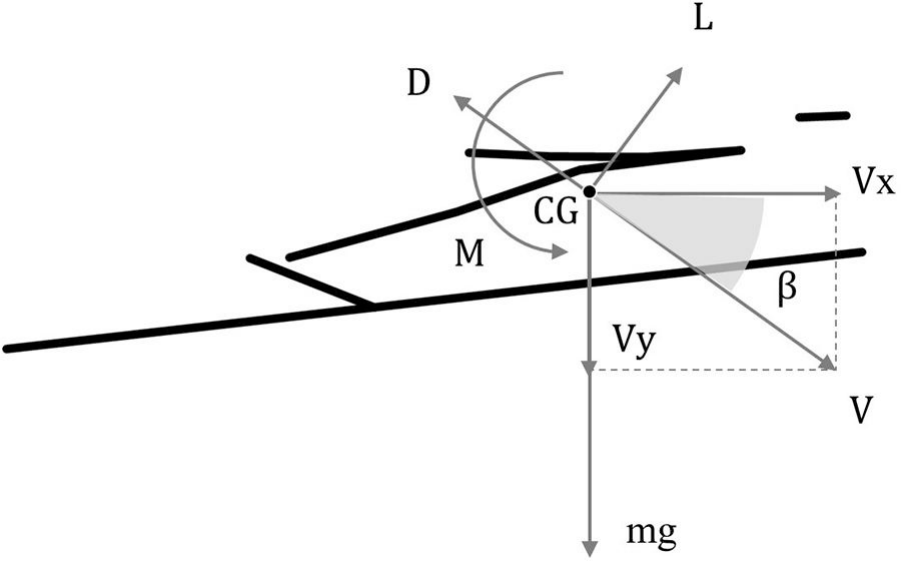
Forces, force components, and the velocity vector acting on the jumper-ski system during the flight phase. V, Velocity vector; *β*, Angle between the velocity vector and the horizontal axis; Vx, Horizontal component of the velocity vector; Vy, Vertical component of the velocity vector; mg, Gravity; L, Lift; D, Drag; M, Moment; CG, Center of gravity of the jumper-ski system.

The forces and force components and the velocity vector acting on the jumper-ski system during the flight phase, are shown in Figure 8. Strategies for increasing the jump distance during the flight phase involve increasing lift and reducing drag. In other words, the flight trajectory is determined by the lift-to-drag ratio. Jumpers must control the lift-to-drag ratio so as not to increase the angle (*β*) between the velocity vector and the horizontal axis. To control the lift-to-drag ratio, jumpers have to control their position. In addition, reducing body mass increases the influence of the lift. The contribution of the flight phase to jump distance increases as hill size increases. In other words, the contribution of the flight phase is lower on smaller hills.

In regard to the lift-to-drag ratio, Gardan et al. (41) showed that it is approximately 0.7 at takeoff and increases to about 1.2 from 1 s after takeoff until landing. Additionally, in actual jumps, the angle of attack at landing is generally around 40° (24).

**Figure 9:**
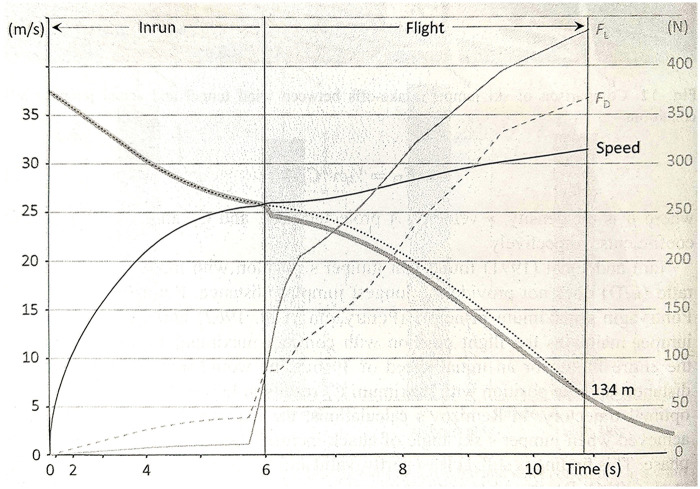
Computer simulation results of velocity, lift, and drag during the approach phase and flight phase for a jump distance of 134 m (42). Inrun, Approach phase; Flight, Flight phase; Speed, Tangential velocity of the jumper-ski system; F_L_, Lift; F_D_, Drag. Reproduced from Virmavirta, 2017 (42) with permission from Springer Nature.

During the flight phase, the velocity of the jumper-ski system increases over time. An example of a large hill is shown. For example, Virmavirta (42) reported that on a large hill, the velocity is approximately 26 m/s at takeoff and increases to approximately 32 m/s at landing. Furthermore, lift and drag are approximately 80 N at takeoff, whereas at landing, the lift increases to approximately 430 N and the drag to approximately 360 N (Figure 9).

#### 4.3.2 Flight position and aerodynamic forces

Several studies on actual jumps have investigated the flight phase (25, 27, 43–45). These studies primarily indicate the importance of smoothly transitioning into flight position immediately after takeoff motion during the early stage of the flight phase. Schwameder et al. (46) and Virmavirta et al. (25) demonstrated that top jumpers could achieve a stable flight state within 0.5 s after takeoff. Furthermore, Arndt et al. (27) reported that approximately 84% of jump distance can be explained by the jumper's position at a point 17 m after takeoff. These results can be explained as a strategy to optimize the lift-to-drag ratio as early as possible, given that the velocity of the jumper-ski system increases over time during the flight phase.

Wind tunnel experiments also demonstrated the relationship between flight position and lift (47). Moreover, computer simulation studies have shown relationships between flight position and jump distance as well as between flight position and aerodynamic forces (24, 41, 48–56). Gardan et al. (41) showed that during the initial stage of flight, the lift-to-drag ratio is not influenced by velocity but depends on the angle of attack and that both lift and drag are strongly affected by changes in the angle of attack. A recent study by Yamamoto et al. (56) demonstrated that top jumpers exhibit higher lift and drag in the early stages of the flight phase compared to other jumpers and maintain a higher lift-to-drag ratio throughout the flight phase. However, in actual jumps, jumpers actively control disturbances in their position caused by crosswinds and other factors during flight. In computer simulations, it is difficult to account for such perturbations in position, which represents a methodological limitation.

In addition, the influence of skis has been shown to be substantial (51), and studies focusing exclusively on skis have been conducted (57, 58). Virmavirta and Kivekäs (57) demonstrated that bindings that keep the ski base horizontal are appropriate.

#### 4.3.3 BMI and body mass

Under the BMI rules, if a jumper's BMI falls below the reference value, the allowable ski length is reduced stepwise. Since some jumpers choose to reduce their body mass even at the expense of ski length, several studies have examined the relationship between BMI or body mass and jump distance (24, 59, 60). Although body mass and jump distance were previously negatively correlated, repeated rule changes have led to the conclusion that the current rules are appropriate, as demonstrated by Virmavirta and Kivekäs (60). Nevertheless, even under the current rules, a certain number of jumpers reduce their body mass despite sacrificing ski length. However, as noted above, the relationship between body mass and approach velocity has become less clear in recent years. If this result also holds true on larger hills, the BMI regulations may be considered appropriate.

#### 4.3.4 Classification of previous studies by hill size

Studies on the flight phase based on actual jumps or conditions similar to actual jumps are classified according to hill size and the results are summarized in Table 4. For wind tunnel experiments and computer simulation studies in which the hill size was not specified, classification was performed based on the wind speed or assumed velocity of the jumper-ski system. The studies assuming velocities of approximately 100 km/h (28 m/s) were classified as flying hills, whereas those assuming velocities of approximately 90 km/h (25 m/s) were classified as large hills. All of these studies corresponded to hills of normal or larger hill size. It should be noted that this classification involves interpretative assumptions. Because there is a variation in hill size within each category, there are limitations in making clear distinctions.

**Table 4:** Main focuses of previous studies in the flight phase and classification by hill size. .

Previous study	Hill Size					Main focus			
	Small hill	Medium hill	Normal hill	Large hill	Flying hill	Relationship between flight position and jump distance	Relationship between flight position and aerodynamic forces	Relationship between BMI or mass and jump distance	Notes
Gardan et al. (41)	No	No	No	Yes	No	No	Yes	No	
Arndt et al. (27)	No	No	Yes	No	No	Yes	No	No	
Virmavirta et al. (25)	No	No	No	Yes	No	Yes	No	No	
Vodičar and Jošt (43)	No	No	No	No	Yes	Yes	No	No	
Jølstad et al. (44)	No	No	No	Yes	No	No	Yes	No	Because the lift-to-drag ratio was calculated using positional data, flight position was not addressed.
Schwameder (46)	No	No	Yes	No	No	Yes	No	No	
Murakami et al.	No	No	No	Yes	No	Yes	No	No	
Seo et al. (47)	No	No	No	Yes	No	No	Yes	No	
Jin et al. (48)	No	No	No	Yes	No	Yes	Yes	No	
Müller et al. (49)	No	No	No	No	Yes	No	Yes	Yes	
Seo et al. (50)	No	No	No	Yes	No	Yes	Yes	No	
Schmölzer and Müller (24)	No	No	No	Yes	No	Yes	Yes	Yes	
Meile et al. (51)	No	No	No	Yes	No	No	Yes	No	
Lee et al. (52)	No	No	No	No	Yes	No	Yes	No	
Jung et al. (53)	No	No	Yes	Yes	Yes	No	Yes	No	
Barnes et al. (54)	No	No	No	Yes	No	Yes	Yes	No	
Hu et al. (55)	No	No	No	No	Yes	No	Yes	No	
Yamamoto et al. (56)	No	No	No	Yes	No	No	Yes	No	
Virmavirta and Kivekäs (57)	No	No	No	No	Yes	No	No	No	This study focused exclusively on skis.
Cao et al. (58)	No	No	No	No	Yes	No	No	No	This study focused exclusively on skis.
Schmölzer and Müller (59)	No	No	No	Yes	No	No	No	Yes	
Virmavirta and Kivekäs (60)	No	No	No	Yes	No	No	No	Yes	

Yes, Indicates that it is subject; No, Indicates that it is not subject.

#### 4.3.5 Studies on rules

Studies focusing exclusively on jump suits worn by jumpers have also been conducted (61–63). Chowdhury et al. (61) suggested that tighter suits increase jump distance. In contrast, rule revisions have been implemented to prevent the excessive looseness of jump suits. Hasegawa et al. (63) suggested that changes in air permeability affect the drag. Based on the assumption that lower air permeability increases jump distance, recent rule changes have restricted suit permeability and jumpers are no longer able to use new suits frequently. However, Hasegawa et al. (63) suggested that higher air permeability may increase the jump distance. Virmavirta et al. (64) investigated the relationships between suit size, air permeability, and jump distance and demonstrated that a larger suit size leads to a longer jump distance, while the effect of air permeability was limited. Elfmark et al. (65) investigated the relationship between jump distance and the use of jump suits of different sizes for actual jumps on large hills. The results showed that jump distance increased as suit size increased. Therefore, the regulations on jump suits used in the World Cup competitions, which are primarily implemented on large hills, are considered appropriate.

During the flight phase, the influence of wind cannot be ignored. Several studies have addressed rules for correcting wind effects (wind factor) (66–70), and there remains room for discussion regarding the methods for calculating the wind factor. However, this is an extremely complex issue. In particular, rule changes related to jump suits are frequent. Although it is clear that reducing suit size decreases the projected area, it is difficult to quantify the magnitude of the effect. Furthermore, the lift and drag are influenced by projected area and air density. Air density is affected by atmospheric pressure, which, in turn, is influenced by air temperature and altitude, adding further complexity to the problem.

### 4.4 Landing phase

#### 4.4.1 Previous studies

In the landing phase, although a lack of studies was pointed out in the 2008 review (6), a more recent review focusing on the landing phase (71) emphasized both the scarcity of and need for further studies.

The landing motion is considered to begin approximately 0.4 s before ground contact, and it has been shown that jumpers who record longer jump distances initiate the landing motion later (72). The landing phase is also influenced by ground effects. Seo et al. (47) addressed these ground effects. It was shown that maintaining the V-style until just before landing increases lift while simultaneously reducing drag.

Studies using insole-type pressure measurement systems have also been conducted (73), showing that the impact force at landing is approximately 2 times body mass. In contrast, Virmavirta and Komi (30) reported that the landing impact is approximately 1.5–3 times the body mass. In addition, when a telemark position is adopted, it has been suggested that the magnitude of the impact acting on the plantar surface may differ between the left and right feet (74). Virmavirta and Komi (30) further demonstrated, using EMG measurements, that lower limb muscle activity increases markedly at the instant of landing.

The telemark position also affected the style points. Some studies have reported relationships between the kinematic variables of telemark position and style points (72).

In recent years, since competitions are operated based on hill size, increasing the jump distance inevitably results in landing on a slope with an inclination closer to the horizontal. Consequently, the impact at landing is also expected to increase. Stenseth et al. (75) reported that the majority of injuries in ski jumping occurred during landing, with the knee being the most frequently injured body part. The potential implications for injury risk should be considered as jumpers rarely change which leg is placed forward in the telemark position.

Studies on the landing phase based on actual jumps or conditions similar to actual jumps are classified according to hill size and the results are summarized in Table 5.

**Table 5:** Main focuses of previous studies in the landing phase and classification by hill size.

Previous study	Hill Size				Main focus				
	Small hill	Medium hill	Normal hill	Large hill	Flying hill	Impact at landing	Landing position	Injury risk	Notes
Greimel et al. (72)	No	No	Yes	No	No	No	Yes	No	
Bessone et al. (73)	No	No	Yes	No	No	Yes	No	No	
Virmavirta and Komi (30)	No	No	Yes	No	No	Yes	No	No	
Bessone et al. (74)	No	No	Yes	No	No	Yes	No	No	
Stenseth et al. (75)	No	No	Yes	Yes	No	No	No	Yes	This is a cohort study rather than a biomechanical study.

Yes, Indicates that it is subject; No, Indicates that it is not subject.

## 5 Perspectives of future studies based on hill size

This review identified gaps in biomechanical knowledge related to increases in jump distance in each phase of the jump.

Based on ski jumping hill design standards, the contributions of the approach and flight phase increase as hill size becomes larger. However, the takeoff phase is different. Specifically, the contribution of the vertical velocity of the CG of the jumper-ski system during the takeoff phase becomes greater as hill size decreases. In other words, as hill size increases, jumpers are required to balance the maximization of the vertical velocity of the CG of the jumper-ski system during the takeoff phase with other biomechanical factors.

As shown in Tables 2–5, previous studies have mainly focused on the normal and large hills that are used in the Olympic Games. Beginners typically start training on small or medium hills and, as their skills improve, they progressively move to larger hills (2). Therefore, findings from studies on small and medium hills are particularly useful for coaching beginners.

When considering each phase separately, a substantial body of knowledge was accumulated for the approach phase, regardless of the hill size. In contrast, biomechanical insights, especially those related to angular momentum, are limited during the takeoff phase. There is a need to accumulate knowledge on angular momentum on medium hills and other hill sizes. In the flight phase, findings related to small and medium hills are also insufficient. In particular, on small hills, training focused on flight position may not always be appropriate. Therefore, studies targeting small- and medium-sized hills are likely to be valuable. In the landing phase, biomechanical knowledge is scarce regardless of the hill size. As noted by Bessone and Schwirtz (71), it is necessary to clarify the relationship between landing mechanics and injuries.

Even for flying hills, which represent the largest hill size and are used in World Cup competitions, the available biomechanical knowledge remains insufficient. A possible reason is the difficulty of data collection; for example, video-based analyses are challenging due to the wide filming range required and presence of approximately 1 m high guards in the approach phase (7). In addition, measuring GRFV requires force plates to be embedded in an actual hill. Moreover, jumpers use different start gate positions according to their individual skill levels in actual jump training. These positions are also frequently adjusted depending on wind conditions, making it difficult to conduct trials under standardized start gate conditions.

In recent years, studies have increasingly employed methods other than video analysis (76, 77). In addition, attempts have been made to estimate forces from video data (78). Therefore, more detailed biomechanical knowledge is expected to be accumulated for various hill sizes in the future.

It is also evident that research on landings is limited. Further studies on the landing phase are required from the perspective of injury prevention.

## 6 Conclusion

This review focuses on biomechanical insights related to the increase in jump distance in ski jumping. The existing findings were organized by phase, and previous studies were classified according to hill size. For each phase, knowledge consistent with the mechanical objectives for increasing jump distance was comprehensively identified. However, when examined by hill size, the majority of available findings were concentrated on normal and large hills, which are used in the Olympic Games. Consequently, the need for future studies on hill sizes other than normal and large hills has become evident. In particular, on large hills, there are constraints on capturing jumper motion. In addition, it is not easy to install equipment such as force plates on large hills. Therefore, for the application of kinematic data to musculoskeletal models and for the estimation of kinetics, the development of methods that enable easier data collection and more accurate kinematic measurements is required. Furthermore, because the research on landing remains insufficient, further studies on the landing phase are needed from the perspective of injury prevention.

## References

[1] Straumann R. Vom Skiweitsprung und seiner Mechanik (1 Teil). Ski Jahrbuch des Schweizerischen SkiVerbandes. (1926) 20:6–29.

[2] Müller E Schwameder H. Biomechanical aspects of new techniques in alpine skiing and ski-jumping. J Sports Sci. (2003) 21:679–92. 10.1080/0264041031000140284 14579866

[3] Janura M Cabell L Elfmark M Vaverka F. Kinematic characteristics of the ski jump inrun: a 10-year longitudinal study. J Appl Biomech. (2010) 26:196–204. 10.1123/jab.26.2.196 20498491

[4] Janurová E Janura M Cabell L Svoboda Z Vařeka I Elfmark M. Kinematic chains in ski jumping in-run posture. J Hum Kinet. (2013) 39:67–72. 10.2478/hukin-2013-0069 24511342 PMC3916932

[5] Schwameder H Müller E. Biomechanics in ski jumping: a review. Eur J Sport Sci. (2001) 1:1–16. 10.1080/17461390100071107

[6] Schwameder H. Biomechanics research in ski jumping 1991–2006. Sports Biomech. (2008) 7:114–36. 10.1080/14763140701687560 18341140

[7] International Ski and Snowboard Federation. The International Ski Competition Rules (ICR), Book III, Ski Jumping. Oberhofen: International Ski and Snowboard Federation FIS (2024). p. 48–52.

[8] Gasser HH. FIS jumping hills, Construction norm 2018. Implementing provisions for art (2018) 411 of the ICR Ski Jumping:5–8 (2018).

[9] Elfmark O Ettema G Gilgien M. Assessment of the steady glide phase in ski jumping. J Biomech. (2022) 139:111139. 10.1016/j.jbiomech.2022.111139 35609493

[10] Virmavirta M Komi PV. Measurement of take-off forces in ski jumping part 2. Scand J Med Sci Sports. (1993a) 3:237–43. 10.1111/j.1600-0838.1993.tb00388.x

[11] Komi PV Nelson RC Pulli M. Biomechanics of Skijumping. Jyväskylä: University of Jyväskylä (1974). p. 7–53.

[12] Virmavirta M Komi PV. The takeoff forces in ski jumping. Int J Sport Biomech. (1989) 5:248–57. 10.1123/ijsb.5.2.248

[13] Virmavirta M Isolehto J Komi PV Schwameder H Pigozzi F Massazza G. Take-off analysis of the Olympic ski jumping competition (HS-106 m). J Biomech. (2009) 42:1095–101. 10.1016/j.jbiomech.2009.02.026 19349050

[14] Vodicar J Jost B. The factor structure of chosen kinematic characteristics of take-off in ski jumping. J Hum Kin. (2010) 23:37–45. 10.2478/v10078-010-0005-6

[15] Elfmark O Ettema G. Aerodynamic investigation of the inrun position in ski jumping. Sports Biomech. (2024) 23:455–69. 10.1080/14763141.2020.1871503 33533308

[16] Zanevskyy I Banakh V. Dependence of ski jump length on the skier’s body pose at the beginning of take-off. Acta Bioeng Biomech. (2010) 12:79–87. 21361260

[17] Ettema GJC Bråten S Bobbert MF. Dynamics of the in-run in ski jumping: a simulation study. J Appl Biomech. (2005) 21:247–59. 10.1123/jab.21.3.247 16260845

[18] Kim D Jung S Lee J. Friction coefficient measurements on jumping ski patterned running surfaces. Tribol Int. (2022) 175:107858. 10.1016/j.triboint.2022.107858

[19] Virmavirta M Komi PV. Kinematics and muscular function in ski jumping. In: Komi PV, editor. Neuromuscular Aspects of Sports Performance XVII. Hoboken: Wiley-Blackwell (2011). p. 91–102.

[20] Müller W. Performance factors in ski jumping. In: Nørstrud H, editor. Sport Aerodynamics. Vienna: Springer (2008). p. 139–60.

[21] Funato Y Nakashima H Sakurai S. Analyzing angular momentum in the takeoff phase of medium-hill ski jumping. Front Sports Act Living. (2025) 7:1643241. 10.3389/fspor.2025.1643241 40959530 PMC12434471

[22] Virmavirta M Komi PV. Ski jumping boots limit effective take-off in ski jumping. J Sports Sci. (2001a) 19:961–8. 10.1080/026404101317108462 11820690

[23] Komi PV Virmavirta M. Ski jumping take-off performance: determining factors and methodological advances. In: Müller E, editor. Science and Skiing. Aachen: Chapman & Hall (1997). p. 3–26.

[24] Schmölzer B Müller W. Individual flight styles in ski jumping: results obtained during Olympic games competitions. J Biomech. (2005) 38:1055–65. 10.1016/j.jbiomech.2004.05.038 15797587

[25] Virmavirta M Isolehto J Komi PV Brüggemann G-P Müller E Schwameder H. Characteristics of the early flight phase in the Olympic ski jumping competition. J Biomech. (2005) 38:2157–63. 10.1016/j.jbiomech.2004.10.004 16154402

[26] Virmavirta M Kivekäs J Komi PV. Take-off aerodynamics in ski jumping. J Biomech. (2001a) 34:465–70. 10.1016/s0021-9290(00)00218-9 11266669

[27] Arndt A Brüggemann GP Virmavirta M Komi PV. Techniques used by Olympic ski jumpers in the transition from takeoff to early flight. J Appl Biomech. (1995) 11:224–37. 10.1123/jab.11.2.224

[28] Yamamoto K Tsubokura M Ikeda J Onishi K Baleriola S. Effect of posture on the aerodynamic characteristics during take-off in ski jumping. J Biomech. (2016) 49:3688–96. 10.1016/j.jbiomech.2016.09.037 27743629

[29] Hu Q Tang W Liu Y. Numerical simulation research on aerodynamic characteristics during take-off phase in ski jumping. Appl Sci. (2024a) 14:1221. 10.3390/app14031221

[30] Virmavirta M Komi PV. Plantar pressures during ski jumping take-off. J Appl Biomech. (2000) 16:320–6. 10.1123/jab.16.3.320

[31] Virmavirta M Perttunen J Komi PV. EMG activities and plantar pressures during ski jumping take-off on three different sized hills. J Electromyogr Kinesiol. (2001b) 11:141–7. 10.1016/s1050-6411(00)00047-x 11228427

[32] Huang Y Jiang L Chen X Sun Q Zhang X Tan X. Musculoskeletal simulation of professional ski jumpers during take-off considering aerodynamic forces. Front Bioeng Biotechnol. (2023) 11:1241135. 10.3389/fbioe.2023.1241135 37720321 PMC10501566

[33] Virmavirta M Koml PV. Measurement of take-off forces in ski jumping. Scandinavian Med Sci Sports. (1993b) 3:229–36. 10.1111/j.1600-0838.1993.tb00387.x

[34] Virmavirta M Komi PV. Kinetics and muscular function in ski jumping. In: Komi PV, editor. Neuromuscular Aspects of Sports Performance XVII. Hoboken, NJ: Wiley-Blackwell (2010). p. 91–102.

[35] Virmavirta M Komi PV. Plantar pressure and EMG activity of simulated and actual ski jumping take-off. Scand J Med Sci Sports. (2001b) 11:310–4. 10.1034/j.1600-0838.2001.110510.x 11696217

[36] Ettema G Hooiveld J Braaten S Bobbert M. How do elite ski jumpers handle the dynamic conditions in imitation jumps? J Sports Sci. (2016) 34:1–7. 10.1080/02640414.2015.1088660 26368027

[37] Pauli CA Keller M Ammann F Hübner K Lindorfer J Taylor WR. Kinematics and kinetics of squats, drop jumps and imitation jumps of ski jumpers. J Strength Cond Res. (2016) 30:643–52. 10.1519/JSC.0000000000001166 26418370 PMC4780482

[38] Lorenzetti S Ammann F Windmüller S Häberle R Müller S Gross M. Conditioning exercises in ski jumping: biomechanical relationship of squat jumps, imitation jumps, and hill jumps. Sports Biomech. (2017) 18:63–74. 10.1080/14763141.2017.1383506 29166832

[39] Ettema G Braatenb S Danielsena J Fjeld BE. Imitation jumps in ski jumping: technical execution and relationship to performance level. J Sports Sci. (2020) 38:2155–60. 10.1080/02640414.2020.1776913 32543286

[40] Ketterer J Gollhofer A Lauber B. Biomechanical agreement between different imitation jumps and hill jumps in ski jumping. Scandinavian Med Sci Sports. (2020) 31:115–23. 10.1111/sms.13834 32969534

[41] Gardan N Schneider A Polidori G Trenchard H Seigneur JM Beaumont F. Numerical investigation of the early flight phase in ski-jumping. J Biomech. (2017) 59:29–3. 10.1016/j.jbiomech.2017.05.013 28558914

[42] Virmavirta M. Ski jumping: aerodynamics and kinematics of take-off and flight. In: Müller B, editor. Handbook of Human Motion. Cham: Springer (2017). p. 1681–701.

[43] Vodičar J Jošt B. The relationship between selected kinematic parameters and length of jumps of the ski-flying competition. Kinesiology. (2011) 43:74–81.

[44] Jølstad PAH Gilgien M Elfmark O. Investigation of individual strategies in the aerial phase in ski jumping. Sci Rep. (2023) 13:22505. 10.1038/s41598-023-49683-0 38110490 PMC10728078

[45] Murakami M Iwase M Seo K Ohgi Y Koyanagi R. High-speed video image analysis of ski jumping flight posture. Sports Eng. (2014) 17:217–25. 10.1007/s12283-014-0157-z

[46] Schwameder H Müller E Lindenhofer E DeMonte G Potthast W Brüggemann P. Kinematic characteristics of the early flight phase in ski-jumping. In: Müller E, editor. Science and Skiing III. Oxford: Meyer and Meyer Sport (2005). p. 381–91.

[47] Seo K Watanabe I Murakami M. Aerodynamic force data for a V-style ski jumping flight. Sports Eng. (2004a) 7:31–9. 10.1007/BF02843971

[48] Jin H Shimizu S Watanuki T Kubota H Kobayashi K. Desirable gliding styles and techniques in ski jumping. J Appl Biomech. (1995) 11:460–74. 10.1123/jab.11.4.460

[49] Müller W Platzer D Schmölzer B. Dynamics of human flight on skis: improvements in safety and fairness in ski jumping. J Biomech. (1996) 29:1061–8. 10.1016/0021-9290(95)00169-7 8817373

[50] Seo K Murakami M Yoshida K. Optimal flight technique for V-style ski jumping. Sports Eng. (2004b) 7:97–103. 10.1007/BF02915921

[51] Meile W Reisenberger E Mayer M Schmölzer B Müller W Brenn G. Aerodynamics of ski jumping: experiments and CFD simulations. Exp Fluids. (2006) 41:949–64. 10.1007/s00348-006-0213-y

[52] Lee KD Park MJ Kim KY. Optimization of ski jumper’s posture considering lift-to-drag ratio and stability. J Biomech. (2012) 45:2125–32. 10.1016/j.jbiomech.2012.05.036 22727524

[53] Jung A Staat M Müller W. Flight style optimization in ski jumping on normal, large, and ski flying hills. J Biomech. (2014) 47:716–22. 10.1016/j.jbiomech.2013.11.021 24388531

[54] Barnes J Tuplin S Walker AD. Flight dynamics of ski jumping: wind tunnel testing and numerical modeling to optimize flight position. Proc Inst Mech Eng P. (2022) 239:193–204. 10.1177/17543371221111625

[55] Hu Q Tang W Liu Y. Computational fluid dynamics simulation study on aerodynamic characteristics under unfavorable conditions during flight phase in ski jumping. Appl Sci. (2024b) 14:1390. 10.3390/app14041390

[56] Yamamoto K Nishino T Bale R Shimada T Miyamoto N Tsubokura M. Numerical study of transient aerodynamic forces acting on a ski jumper considering dynamic posture change from takeoff to landing. Sports Biomech. (2022) 24:763–77. 10.1080/14763141.2022.2154256 36510445

[57] Virmavirta M Kivekäs J. Aerodynamics of an isolated ski jumping ski. Sports Eng. (2019a) 22:8. 10.1007/s12283-019-0298-1

[58] Cao L Guo Y Li X Chen L Wang X Zhao T. Optimization of ski attitude for the in-flight aerodynamic performance of ski jumping. Biology (Basel). (2022) 11:1362. 10.3390/biology11091362 36138841 PMC9495398

[59] Schmölzer B Müller W. The importance of being light: aerodynamic forces and weight in ski jumping. J Biomech. (2002) 35:1059–69. 10.1016/S0021-9290(02)00066-0 12126665

[60] Virmavirta M Kivekäs J. Is it still important to be light in ski jumping? Sports Biomech. (2019b) 20:407–18. 10.1080/14763141.2018.1556326 30689521

[61] Chowdhury H Alam F Mainwaring D. Aerodynamic study of ski jumping suits. Procedia Eng. (2011a) 13:376–81. 10.1016/j.proeng.2011.05.101

[62] Chowdhury H Moria H Alam F Subic A. Aerodynamics of ski jumping suits. Sports Technol. (2011b) 4:164–70. 10.1080/19346182.2012.725411

[63] Hasegawa H Kawabata Y Murakami M Kazuya SEO. Shigeru obayashi, effect of air permeability on the aerodynamic characteristics of ski jumping suits. Adv Exper Mech. (2018) 3:118–22. 10.11395/aem.3.0_118

[64] Virmavirta M Müller S Kürschner M Bessone V Krężałek P Elfmark O. Influence of suit size and air permeability on performance in ski jumping. Part I: wind tunnel measurements. Front Sports Act Living. (2025) 7:1693699. 10.3389/fspor.2025.1693699 41245645 PMC12611860

[65] Elfmark O Müller S Kürschner M Bessone V Krężałek P Virmavirta M. The influence of suit size on performance in ski jumping. Part II: field measurements. Front Sports Act Living. (2026) 8:1693723. 10.3389/fspor.2026.1693723 41769159 PMC12945395

[66] Jung A Müller W Staat M. Wind and fairness in ski jumping: a computer modelling analysis. J Biomech. (2018) 75:147–53. 10.1016/j.jbiomech.2018.05.001 29803308

[67] Jung A Müller W Staat M. Optimization of the flight technique in ski jumping: the influence of wind. J Biomech. (2019) 88:190–3. 10.1016/j.jbiomech.2019.03.023 30940358

[68] Jung A Müller W Virmavirta M. A heuristic model-based approach for compensating wind effects in ski jumping. J Biomech. (2021) 125:110585. 10.1016/j.jbiomech.2021.110585 34233216

[69] Virmavirta M Kivekäs J. The effect of wind on jumping distance in ski jumping—fairness assessed. Sports Biomech. (2012) 11:358–69. 10.1080/14763141.2011.637119 23072046

[70] Mikko V Juha K. The effect of wind on jumping distance in ski jumping depends on jumpers’ aerodynamic characteristics. J Biomech. (2022) 137:111101. 10.1016/j.jbiomech.2022.111101 35490447

[71] Bessone V Schwirtz A. Landing in ski jumping: a review about its biomechanics and the connected injuries. J Sci Sport Exerc. (2021) 3:238–48. 10.1007/s42978-020-00096-9

[72] Greimel F Virmavirta M Schwameder H. Kinematic analysis of the landing phase in ski jumping. In: Müller E Lindinger S Stöggl T, editors. Science and Skiing IV. Aachen: Meyer and Meyer Sport (2009). p. 721–7.

[73] Bessone V Petrat J Schwirtz A. Ski position during the flight and landing preparation phases in ski jumping detected with inertial sensors. Sensors. (2019a) 19:2575. 10.3390/s19112575 31174278 PMC6603655

[74] Bessone V Petrat J Schwirtz A. Ground reaction forces and kinematics of ski jump landing using wearable sensors. Sensors. (2019b) 19:2011. 10.3390/s19092011 31035683 PMC6539877

[75] Stenseth OMR Barli SF Martin RK Engebretsen L. Injuries in elite women’s ski jumping: surveillance through the 2017–18 FIS world cup season. Br J Sports Med. (2020) 54:44–8. 10.1136/bjsports-2019-100799 31527043 PMC6923946

[76] Elfmark O Ettema G Groos D Ihlen EAF Velta R Haugen P. Performance analysis in ski jumping with a differential global navigation satellite system and video-based pose estimation. Sensors. (2021) 21:5318. 10.3390/s21165318 34450758 PMC8399095

[77] Yu J Ma X Qi S Liang Z Wei Z Li Q. Key transition technology of ski jumping based on inertial motion unit, kinematics and dynamics. Biomed Eng OnLine. (2023) 22:21. 10.1186/s12938-023-01087-x 36864414 PMC9983218

[78] Nam Y Do Y Kim J Lee H Kim DN. A hybrid framework to predict ski jumping forces by combining data-driven pose estimation and model-based force calculation. Eur J Sport Sci. (2023) 23:221–30. 10.1080/17461391.2022.2028013 35001852

